# Is Periodontal Inflammation Associated with Liver Cirrhosis? A Cross-Sectional Study

**DOI:** 10.3390/jcm14186616

**Published:** 2025-09-19

**Authors:** Goran Rinčić, Marija Roguljić, Nives Rinčić, Lucija Virović Jukić, Petar Gaćina, Darko Božić, Ana Badovinac

**Affiliations:** 1 Sestre Milosrdnice University Hospital Centre, Division of Hematology, Department of Internal Medicine, University of Zagreb School of Dental Medicine, 10000 Zagreb, Croatia; grincic@yahoo.com (G.R.); petar.gacina@kbcsm.hr (P.G.); 2 Department of Periodontology, School of Medicine, University of Split, 21000 Split, Croatia; 3 Department of Periodontology, Dental Outpatient Clinic Zagreb, 10000 Zagreb, Croatia; marijarog@gmail.com; 4 Division of Gastroenterology and Hepatology, Department of Internal Medicine, Sestre Milosrdnice University Hospital Centre, 10000 Zagreb, Croatia; lucija.virovic.jukic@kbcsm.hr; 5 Department of Periodontology, School of Dental Medicine, University of Zagreb, 10000 Zagreb, Croatia; bozic@sfzg.hr (D.B.); badovinac@sfzg.hr (A.B.)

**Keywords:** periodontitis, inflammation, severe periodontitis, liver cirrhosis, alcohol abuse, systemic disease

## Abstract

**Background**: Periodontitis is linked to a range of systemic non-communicable diseases, including hepatic diseases. The aim of this study was to investigate whether periodontal health status is associated with liver cirrhosis (LC). **Methods**: Patients were recruited from the Department of Internal Medicine at the University Clinical Hospital “Sestre Milosrdnice” and categorized into two groups. The case group comprised patients with LC, while age-matched individuals without LC served as controls. Systemic health status was evaluated through laboratory tests, medical history, and clinical parameters, and the Model for End-Stage Liver Disease (MELD) score was calculated for each participant. A comprehensive clinical periodontal assessment was conducted, measuring bleeding on probing (BoP), probing pocket depth (PPD), gingival recession (GR), clinical attachment level (CAL), and the Periodontal Inflamed Surface Area (PISA) score. Stepwise logistic regression was employed to assess possible predictors of LC, including periodontal status. **Results**: A total of 100 patients were included in the analysis, consisting of 50 cases with LC and 50 controls. The mean age was 56.79 years (SD = 11.16) of participants, and 58% were male. The majority of LC cases were attributed to alcohol abuse (41/50, 82%), and the median MELD score was 16 (IQR 9–21). Comparison of the two groups revealed significantly worse clinical periodontal parameters in the LC group and a higher prevalence of periodontitis (*p* = 0.012). Among the 50 LC patients, 46 (92%) exhibited severe forms of periodontitis (stages III and IV). Logistic regression analysis identified alcohol consumption (OR = 275.0, 95% CI 52.8–1432.9, *p* < 0.001) and PISA (OR = 28.3, 95% CI 8.3–96.8, *p* < 0.001) as independent predictors of LC. **Conclusions**: Within the limits of the present study, the higher prevalence of periodontal disease in the LC group suggests an association between LC and periodontitis.

## 1. Introduction

Periodontitis is associated with a range of systemic non-communicable diseases, including metabolic, cardiovascular, neurological, pulmonary, and hepatic diseases [1,2,3]. Severe forms of periodontitis are among the most common oral diseases caused by biofilm dysbiosis and an inadequate immune response [4,5]. In moderate to severe forms of periodontitis, the surface of the inflamed pocket epithelium reaches the size of a palm and allows a vast number of bacteria and/or their toxins to enter the bloodstream, which may then cause a systemic pro-inflammatory status [6]. Its association with a number of systemic diseases could be explained by chronic inflammation and a dysregulated immune response [7,8]. Additionally, periodontitis and these systemic conditions share common risk factors, such as genetic predisposition, advanced age, and lifestyle habits that contribute to their onset and progression [9]. These factors increase susceptibility and accelerate the progression of comorbidities [10].

Chronic liver diseases (CLDs) encompass a variety of aetiologies, including alcohol abuse, viral infections, genetic and metabolic disorders, and autoimmune diseases [11]. Liver cirrhosis (LC) represents the advanced stage of CLD and is characterized by a progressive loss of functional hepatic tissue and its replacement with non-functional regenerative nodules encased in fibrous tissue. This pathological transformation leads to several complications, including anaemia, leukopenia, thrombocytopenia, and impaired synthesis of proteins and clotting factors, along with the development of life-threatening conditions [3,11]. Thus, LC is a significant public health problem and is associated with 4% of global deaths [11]. Among the well-known risk factors of morbidity and mortality are the common complications of LC: variceal bleeding, hepatorenal syndrome, hepatic encephalopathy, hepatocellular carcinoma, and infections [12,13].

While numerous studies have explored the association between periodontitis and common chronic non-communicable diseases such as diabetes and cardiovascular diseases, research on the possible relationship between LC and periodontitis is limited [14]. The available literature suggests that periodontitis may be associated with the progression and worse outcomes of liver disease through dysbiosis of oral bacteria [15]. It may be hypothesized that patients with LC have worse periodontal health compared to healthy controls. Therefore, the aim of this study was to investigate the periodontal health status in patients with LC.

## 2. Materials and Methods

### 2.1. Study Design and Setting

This cross-sectional study was conducted at the Department of Periodontology, School of Dental Medicine, University of Zagreb, and at the Division of Haematology, Department of Internal Medicine of the University Hospital “Sestre Milosrdnice” in Zagreb, Croatia, between December 2021 and June 2023, in accordance with the STROBE checklist [16] and the Declaration of Helsinki [17].

The study protocol was approved by the Ethics Committees of the University Hospital “Sestre Milosrdnice” (EP-18031/19-5) and the School of Dental Medicine, University of Zagreb (No. 05-PA-30-X-10/2019).

All participants provided written informed consent before entering the study.

### 2.2. Participants

Our sample consisted of consecutive adult patients recruited at the Department of Internal Medicine of the University Hospital “Sestre Milosrdnice” diagnosed with LC according to clinical and laboratory findings were recruited for this study as a test group. Since the scope of the study was to investigate periodontal inflammation among the patients with LC, we included only the patients with 15 or more teeth to estimate properly supportive periodontal tissues. Age-matched patients without liver cirrhosis were included in the control group. The control group patients were individuals hospitalized due to diagnoses completely unrelated to liver cirrhosis, and in all cases, liver cirrhosis was excluded by laboratory, clinical, and ultrasound examination. The control group patients had normal liver function tests and no history of liver disease or known risk factors for liver disease including: excessive alcohol consumption (defined as >20 g alcohol/day for women and >30 g alcohol/day for men), known metabolic risk factors for MASLD (arterial hypertension, diabetes mellitus overweight/obesity, arterial hypertension of dyslipidemia) history of i.v. drug use or blood transfusions before 1993, history of autoimmune disease, family history of liver disease, or other significant comorbidities requiring chronic therapy. They were not taking hepatotoxic medications. All patients who agreed to participate in the study were referred to the Department of Periodontology, School of Dental Medicine, University of Zagreb, where they were given detailed information about the research protocol, provided informed consent, and underwent a comprehensive clinical periodontal examination.

The exclusion criteria for all participants were having fewer than 15 teeth, being pregnant or lactating, having comorbidities such as diabetes mellitus and arterial hypertension (AH), history of periodontal treatment, or antibiotic use during the past 6 months. Those who declined participation were also excluded.

**Figure 1:**
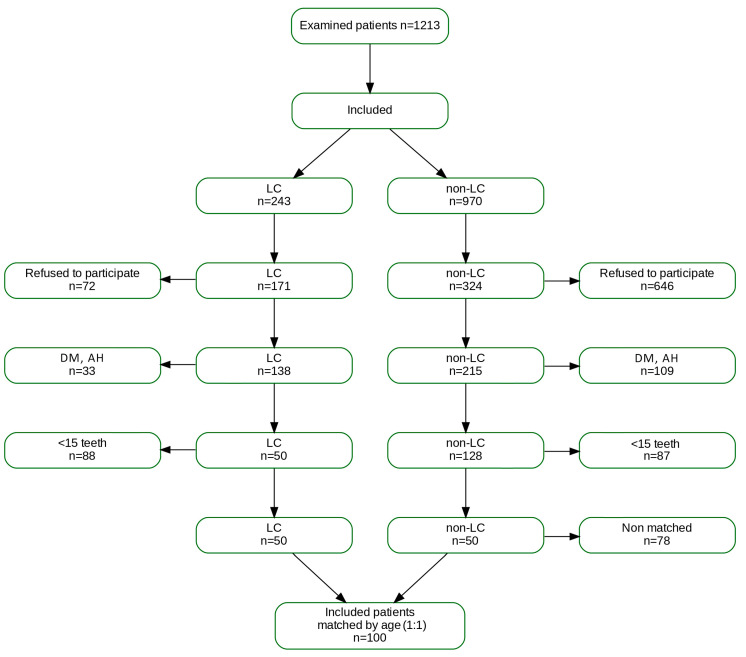
Flow diagram of patient recruitment.

Finally, we included 100 patients, 50 with liver cirrhosis and 50 without. The test group included was created according to the defined inclusion and exclusion criteria, and the control group was accurately age-matched with the test group (Figure 1).

### 2.3. Clinical Medical Data and Assessment

#### 2.3.1. Parameters from Medical History

The referring physicians who enrolled patients for periodontal examination were blinded to the periodontal diagnosis.

Data derived from medical records included age, gender, level of education (number of school years), annual income (in euros), lifestyle habits (oral hygiene, tobacco and alcohol use), body weight (kg), height (cm), and body mass index (BMI). The aetiology of cirrhosis was also recorded.

#### 2.3.2. Laboratory Parameters

Laboratory tests included serum concentrations of albumin (g/L), bilirubin (mg/dL), alkaline phosphatase, aminotransferases (AST, ALT) (U/L), and γ-glutamyl transferase (GGT) (U/L), as well as prothrombin time (%). All laboratory tests were performed at the same laboratory and extracted from the participants’ medical records.

Laboratory parameters such as MELD (Model for End-Stage Liver Disease) and Child–Pugh scores were extracted for test group participants.

The MELD score was used to assess the severity of CLD and to predict mortality risk in patients with LC. It was calculated based on serum bilirubin (mg/dL), creatinine (mg/dL), and INR (International Normalized Ratio) using the following formula:

MELD = 3.78 × ln[bilirubin] + 11.2 × ln[INR] + 9.57 × ln[creatinine] + 6.43

The MELD score typically ranges from 6 to 40, with higher scores indicating more severe liver dysfunction [18].

The Child–Pugh score was also extracted to assess the severity of LC. It includes clinical (ascites, hepatic encephalopathy) and laboratory (bilirubin, serum albumin, prothrombin time) parameters reflecting liver function. The system classifies patients into three categories: Class A (mild), Class B (moderate), and Class C (severe liver disease) [19,20].

### 2.4. Clinical Periodontal Assessment

One experienced and precalibrated periodontist (NR) performed all periodontal examinations using a UNC-15 periodontal probe (Aesculap, Tuttlingen, Germany). The examiner was blinded for patients’ medical diagnosis. The total number of teeth was recorded, and clinical parameters were evaluated at 6 sites per tooth (mesiobuccal, buccal, distobuccal, mesiolingual, lingual, distolingual). Intraexaminer Reproducibility was achieved by a calibration exercise to obtain acceptable reproducibility for PD and recession of the gingival margin. Ten patients, each with 10 teeth (single and multirooted) with PD > 6 mm on at least one aspect of each tooth, were used to calibrate the examiner. The examiner evaluated the patients on two occasions, 48 h apart. Calibration was accurate if measurements at baseline and 48 h were the same at >90% of sites.

The plaque index (PI) [21] and bleeding on probing (BoP) were recorded dichotomously (present/absent), and results were expressed as percentages (number of sites with plaque/bleeding divided by total sites). Probing pocket depth (PPD), gingival recession (GR), and clinical attachment level (CAL) were measured in millimeters. CAL was calculated by combining PPD and GR. According to the current classification of periodontal diseases [22], patients were categorized as with or without periodontitis and staged as I–IV. Severe periodontitis was defined as Stage III or IV, and mild to moderate as Stage I or II.

#### PISA—Periodontal Inflamed Surface Area (PISA)

Based on CAL, GR, and BoP, the PESA (Periodontal Epithelial Surface Area) and PISA (Periodontal Inflamed Surface Area) scores were calculated according to Nesse et al. [23]. PISA represents the surface area of inflamed periodontal tissue, serving as a quantitative measure of systemic inflammatory burden.

### 2.5. Statistical Analyses

A power analysis based on a previous study [24] indicated that a sample of 100 participants (50 per group) was needed to detect significant differences with 90% power and α = 0.05. For periodontitis prevalence, the estimated effect size was 0.42. A χ^2^ goodness of fit test for a 2 × 2 contingency table showed that a total of 100 subjects evenly split between the cirrhosis and the control group would achieve approximately 92% power at a 0.05 significance level. The effect size for BoP was estimated at only 0.28. With such a small effect, about 200 subjects per group would be needed to reach 80% power. The number of sites with pocket depth 4–6 mm and >6 mm yielded much larger effect sizes, amounting to 0.70 and 1.22, respectively. Using parametric tests with an allocation ratio of 1, the sample size of 50 participants per group was sufficient to achieve statistical power above 95% and 98%, respectively. Therefore, a total sample size of 100 subjects was chosen and divided equally between the cirrhosis and the control group, as a pragmatic compromise that enables approximately 90% power for the pocket depth related outcomes, while acknowledging that BoP remained underpowered at this sample size.

Categorical variables were expressed as frequencies and percentages. Normality of numerical variables was tested with the Shapiro–Wilk test. Normally distributed data were presented as mean ± standard deviation (SD), and non-normal data as median and interquartile range (IQR). Between-group comparisons used the chi-square test for categorical variables and either the T-test or Mann–Whitney U test depending on normality. Also, we conducted logistic regression analysis that included PISA score, age, gender, BMI, smoking, alcohol consumption, and socioeconomic status. Linear regression on logit transformed dependent variable; in order to make linearization efficient we also transformed continuous independent variables (i.e., age, BMI and PISA score were made ordinal by discretizing them into quartiles. Furthermore, a variable selection using LASSO was performed on such model, and none of the variables were found to be superfluous. Finally, model coefficients and their 95%CI were estimated by OLS with HC1 robust standard errors.

Statistical significance was set at *p* < 0.05. All analyses were performed using JASP software (v0.8.5.1; JASP Team, Amsterdam, The Netherlands, 2017) and GRETL: Econometric Software for the GNU Generation (accessed: 4 September 2025 http://www.jstor.org/stable/30035190).

## 3. Results

A total of 1213 patients were screened, and 100 patients were recruited—50 with liver cirrhosis (test group) and 50 without (control group).

Among the 100 participants, 58 (58%) were men, and the mean age was 56.79 years (SD = 11.16). The majority of LC cases had alcohol-related cirrhosis (41 [82%]), were less educated (*p* = 0.002), and had lower socioeconomic status (*p* = 0.02) compared to controls (Table 1).

Statistically significant differences were observed in all clinical periodontal parameters except for the number of teeth (*p* = 0.433). The PISA score was significantly higher in LC cases compared to non-LC controls (*p* < 0.001). Periodontitis (*p* = 0.012), particularly severe forms (*p* < 0.001), was more prevalent in the LC group (Table 2).

All laboratory parameters relevant to the diagnosis of LC were significantly elevated in the LC group (*p* < 0.01). The median MELD score in the LC group was 16 and it was not calculated for the control group (Table 3). Child–Pugh scores further confirmed the severity of disease: 17/50 (34%) were categorized as Class A, 14/50 (28%) as Class B, and 19/50 (38%) as Class C.

Logistic regression analysis based on known risk factors and our hypothesis was made. It included age, gender, BMI, socio economic status, smoking (no, up to 20 cig/day, >20 cig/day) (no drinks, up to 6 drinks/week, more than 6 drinks/week), alcohol consumption and PISA score. Model intercept was −12.6 ± 2.83, adj. R^2^ = 64%, *p* < 0.001 (F test). (Table 4).

## 4. Discussion

The present study comparatively investigated the clinical periodontal and medical parameters in a group of participants with or without liver cirrhosis. The findings in the age test group indicated significantly worse periodontal health and more severe forms of periodontitis in the LC group. The PISA score was found to be an independent predictor of LC, along with alcohol consumption.

Bacterial infections in patients with LC may cause life-threatening complications and are regarded as a major cause of death [11,13]. The present findings are in line with a previous study that reported a higher prevalence of severe periodontitis in patients with LC [25]. Periodontitis is a chronic inflammatory disease for which the major etiological factor is pathogenic bacteria in microbial dental plaque. Numerous studies have reported increased circulating levels of C-reactive protein (CRP) in patients suffering from severe periodontitis [6,26]. Apart from the severe inflammation in periodontal tissues, periodontitis may cause a systemic inflammation reflected by increased CRP and immunoglobulin concentrations in the blood circulation [26]. Bacteria themselves and/or their toxins have been detected in blood samples of patients with severe periodontitis, possibly linking periodontitis with various systemic diseases and conditions [27].

The current classification of periodontitis involves the number of teeth lost due to periodontitis [22]. However, the true cause of tooth loss may not be known in many cases. Moreover, the number of teeth affected by periodontitis determines the surface area of inflamed periodontal soft tissue, and this area decreases significantly when teeth are lost [28]. In accordance with the findings of a prospective study by Ladegaard Grønkjær et al. [28] stating that the burden of inflammation decreases with tooth loss, we included individuals having at least 15 teeth. The study showed that severe periodontitis was associated with higher cirrhosis mortality, whereas mortality was not associated with edentulism. Furthermore, in a retrospective study by Aberg et al. [29], it was shown that dental infections, including severe periodontitis, accelerated the progression of liver diseases as estimated with the MELD score. Accordingly, Ladegaard Grønkjær et al. [28] found a more favourable MELD score in edentulous patients compared to patients with periodontitis, providing further support for the impact of periodontal inflammation on systemic conditions. In line with the previous reports, all laboratory parameters for establishing the diagnosis of LC were significantly higher among cases, indicating liver tissue damage and impaired function. The MELD score was 16 in the group with LC, indicating an increased risk of mortality and the need for liver transplantation [30].

Cirrhotic patients are more susceptible to bacterial infections due to the impairment of both innate and adaptive immune responses, which probably contributes to the onset and progression of periodontitis [3]. Neutrophil dysfunction [31,32], reduced number and dysfunction of monocytes [33], T lymphocytes [34], and natural killer cells [35], acting in the etiopathogenesis of LC, may explain the possible association between periodontitis and LC [24,36,37]. Nagao and Tanigawa showed that the red complex periodontal bacteria were strongly associated with LC of viral etiology [36], also indicating the role of periodontal inflammation in the pathogenesis and development of LC. The present findings provide further support for the possible contribution of periodontal inflammation to the development of LC which is line with data from the literature [24,37]. Considering the present findings together with those of the previous clinical studies that showed negative impact of alcohol abuse on risk of periodontitis as well as increased systemic inflammation in patients with periodontitis [24,37], a bidirectional relationship may be suggested between periodontitis and LC. However, future studies are needed to better clarify this issue.

Along with tobacco smoking and dietary habits, alcohol consumption is recognized as a modifiable lifestyle risk factor affecting both systemic and oral health. Alcohol use disorder is a major public health problem, as it is the third leading cause of death worldwide, following malignant and cardiovascular diseases [38]. Alcohol dependence is strongly associated with the development of LC, increasing associated complications and mortality rates in affected individuals [12]. Alcohol addiction has also been suggested to be a potential risk factor for periodontitis, although the evidence is limited [9,39]. The present findings clearly showed the association of LC with alcohol addiction, probably negatively influencing behaviour and motivation of patients, as reflected in poorer oral hygiene habits. Exploring the influence of alcohol on LC or periodontitis was beyond the scope of the present study; however, the findings suggest that individuals addicted to alcohol have a higher risk of developing not only LC but also severe forms of periodontitis. It could be concluded that alcohol abuse represents a potential mechanistic link that amplifies these two conditions.

According to the present findings, patients with LC had significantly lower education levels and lower socioeconomic status, which probably resulted in poor oral hygiene habits and greater dental plaque accumulation. These findings are in line with a previous study reporting an association between lower levels of education, socioeconomic status, increased alcohol consumption, and periodontitis severity [40]. Interestingly, it has been shown that individuals consuming higher amounts of alcohol are more likely to have higher plaque scores even when they maintain regular daily toothbrushing habits [41,42]. Thus, complex mechanisms are possibly acting along the long-term alcohol consumption/hazardous effects axis, with a particular focus on immune response and increased susceptibility to bacterial infections [39]. This patient population should be advised on the benefits of periodontal treatment, with particular emphasis on plaque control, which can be further improved through the use of antibacterial mouth rinses in addition to tooth brushing [43]. Periodontitis is commonly neglected and underestimated among patients with severe systemic chronic diseases. However, the growing evidence emphasizes its importance not only for oral health but also for the general well-being of individuals [10]. Maintenance of appropriate home care plays a significant role in the prevention of periodontitis, and contemporary treatment protocols provide excellent treatment outcomes. Non-surgical periodontal treatment protocols aim to resolve periodontal inflammation, improve awareness on controlling risk factors, and ensure long-term stability of tooth-supporting tissues [44]. Evidence shows that such as *Aggregatibacter actinomycetemcomitans*, *Prevotella intermedia*, *Fusobacterium necrophorum* *Porphyromonas gingivalis*, *Tannerella forsythia* and *Treponema denticola* are more prevalent in LC patients [29,36]. It could be concluded that periodontal infections may influence a clinical course in this patient population. A recently published review on the current concepts regarding LC and periodontitis suggests that patients with LC should be routinely screened and receive timely treatment for periodontal disease [15]. Periodontal therapy may help reduce liver-derived inflammatory damage and could even have an impact on lowering mortality rates [15]. Closer collaboration between oral healthcare providers and physicians treating gastroenterological disorders may therefore improve both oral and general health outcomes. However, future interventional studies are needed to clarify the potential impact of periodontal treatment, as well as behavioural and lifestyle changes, on chronic liver disease (CLD) and LC in various clinical settings.

The present study has several limitations.

Due to its cross-sectional design, the study findings are limited to demonstrating an association between periodontitis and LC, without the ability to infer causality.

Additionally, the consecutive sampling method used for patient recruitment may involve selection bias, despite the application of strict inclusion criteria. However, the control group did not include generally healthy individuals since participants were recruited from the Department of Internal Medicine that might impact the study results. Furthermore, this study focused only on lifestyle risk factors and did not analyse participants’ comorbidities, which may represent a potential confounding factor. Future research should incorporate other prevalent chronic diseases alongside LC to provide a more comprehensive assessment. The sample in our study may not fully represent the broader population, which limits the generalizability of the findings. Including only patients having at least 15 teeth reduced the group sizes and may have underestimated the true prevalence of periodontitis, as severe periodontitis continues to be one of the most common reasons for tooth loss in the adult population.

## 5. Conclusions

In conclusion, within the limitations of this cross-sectional study, periodontal inflammation could be associated with liver cirrhosis. The findings emphasize the importance of periodontal health and underline the need for close collaboration between oral and systemic healthcare providers. Future prospective and interventional studies are warranted to further explore the bidirectional relationship between periodontitis and liver diseases.

## Figures and Tables

**Table 1:** General characteristics of participants according to diagnosis of liver cirrhosis.

Variable	All (N = 100)	Non-Cirrhosis (N = 50)	Cirrhosis (N = 50)	*p*
Age (mean, SD)	56.79 (11.16)	55.58 (10.797)	58.00 (11.49)	0.280
Gender (male), n (%)	58 (58)	24 (48)	34 (68)	**0.043**
Gender (female), n (%)	42 (42)	26 (52)	16 (32)	
Body mass (median, IQR)	80 (67.75–89)	81.5 (69.25–89.75)	80 (66.25–86.25)	0.426
Height (mean, SD)	1.74 (0.10)	1.75 (0.10)	1.72 (0.09)	0.191
BMI (median, IQR)	25.83 (22.99–30.11)	26.31 (23.62–30.45)	25.83 (22.91–28.12)	0.657
Level of education 8 years n (%)	11 (11)	0 (0)	11(22)	**0.002**
Level of education 12 years n (%)	63 (63)	34 (68)	29 (58)	
Level of education 16 years n (%)	26 (26)	16 (32)	10 (20)	
No income n (%)	10 (10)	3 (6)	7 (14)	**0.020**
Income < 700€ n (%)	43 (43)	16 (32)	27 (54)	
Income 700–1300€ n (%)	38 (38)	24 (48)	14 (28)	
Income >1300€ n (%)	9 (9)	7 (14)	2 (4)	
OH habits < 1/day	6 (6)	2 (4)	4 (8)	**0.002**
OH habits 1/day	33 (33)	9 (18)	24 (48)	
OH habits 2/day	61 (61)	39 (78)	22 (44)	
No smoking n (%)	60 (60)	34 (68)	26 (52)	0.211
Smoking: < 20/day n (%)	33 (33)	14 (28)	19 (38)	
Smoking: >20/day n (%)	7 (7)	2 (4)	5 (10)	
No alcohol n (%)	51 (51)	41 (82)	10 (20)	**<0.001**
Alcohol: < 6 drinks/week n (%)	16 (16)	9 (0)	7 (14)	
Alcohol: >6 drinks/week n (%)	33 (33)	0 (0)	33 (66)	
No cirrhosis n (%)	50 (50)	50 (100)	0 (0)	**<0.001**
Cirrhosis with alcohol n (%)	41 (41)	0 (0)	41 (82)	
Cirrhosis with no alcohol n (%)	9 (9)	0 (0)	9 (18)	

Abbreviations: SD—standard deviation; IQR—interquartile range; BMI—Body Mass Index; OH—Oral Hygiene. Chi-square test was used for categorical variables, *t*-test for normally distributed numerical variables, and Mann–Whitney U test for numerical variables which showed deviation from normality. Statistically significant values (*p* < 0.05) are bolded.

**Table 2:** Periodontal status of participants.

Variable	All (N = 100)	Non-Cirrhosis (N = 50)	Cirrhosis (N = 50)	*p*
Number of teeth median (IQR)	24 (19–27)	24 (19–26)	24 (19–28)	0.433
Plaque index (PI), median (IQR)	82.66 (70.95–93.39)	76.27 (69.5–84.82)	87.73 (75.42–95.6)	**0.003**
Bleeding on probing (BoP), median (IQR)	57.61 (46.28–71.71)	48.77 (43.88–57.48)	68.75 (59.96–82.01)	**<0.001**
Average probing depth (PD, mm), median (IQR)	3.12 (2.79–3.64)	2.980 (2.78–3.12)	3.635 (3.027–4.24)	**<0.001**
Average total CAL (mm), median (IQR)	4.46 (3.88–5.29)	4.05 (3.54–4.43)	5.01 (4.54–6.09)	**<0.001**
PESA, median (IQR)	1555.39 (1246.4–1908.74)	1348.915 (1159.71–1648.76)	1827.8 (1470.46–2229.54)	**<0.001**
PISA, median (IQR)	972.16 (681.87–1310.81)	710.235 (496.66–932.17)	1308.270 (1078.89–1691.51)	**<0.001**
Periodontitis: no n (%)	6 (6)	6 (12)	0 (0)	**0.012**
Periodontitis: yes n (%)	94 (94)	44 (88)	50 (100)	
Periodontitis stage I, II n (%)	50 (50)	46 (92)	4 (8)	**<0.001**
Periodontitis stage III, IV n (%)	50 (50)	4 (8)	46 (92)	

Abbreviations: IQR—interquartile range; CAL—Clinical Attachment Level; PESA—Periodontal Epithelial Surface Area; PISA—Periodontal Inflamed Surface Area. Chi-square test was used for categorical variables; Mann–Whitney U test was used for numerical variables. Statistically significant values (*p* < 0.05) are bolded.

**Table 3:** Diagnostic clinical and laboratory parameters of liver cirrhosis.

Variable, Median (IQR)	All (N = 100)	Non-Cirrhosis (N = 50)	Cirrhosis (N = 50)	*p*
MELD	NA	NA	16 (9–21)	NA
Alkaline phosphatase	83.5 (61.75–128)	65 (56.5–82)	115.5 (86.25–144.5)	**<0.001**
AST	26 (19–41.75)	19.5 (16.25–25)	42.5 (29–73.5)	**<0.001**
ALT	23 (15.75–35.25)	19.5 (15–25.75)	24 (18.25–39.25)	**0.012**
GGT	33 (20.75–74.25)	21.5 (16–33)	56.5 (36.25–188)	**<0.001**
Bilirubin total	13 (8–32.5)	9 (7–12)	32.5 (14.75–82.75)	**<0.001**
Albumin (g/L)	38 (29–42)	41 (37.25–43)	32 (25.33–38.75)	**<0.001**
Prothrombin time (%)	99 (63.5–116.25)	116 (105.25–121)	63 (46.25–82.5)	**<0.001**

Abbreviations: IQR—interquartile range; MELD—Model for End-Stage Liver Disease; AST—Aspartate aminotransferase; ALT—Alanine aminotransferase; GGT-γ—glutamyl transferase. Mann–Whitney U test. Statistically significant values (*p* < 0.05) are bolded.

**Table 4:** Logistic regression model evaluating the variables associated with LC.

Variable		95%CI			95%CI		
	β	Low	hi	OR	Low	hi	*p*
Gender (F)	1.5	−0.8	3.9	4.6	0.4	48.4	0.209
Age	1.0	−0.1	2.1	2.7	0.9	8.3	0.080
BMI	−0.9	−1.8	0.0	0.4	0.2	1.0	0.051
Socio-econom. status	−0.3	−1.6	0.9	0.7	0.2	2.4	0.579
Smoking	−1.2	−3.1	0.8	0.3	0.0	2.2	0.244
Alcohol consumption	5.6	4.0	7.3	275.0	52.8	1432.9	<0.001
PISA	3.3	2.1	4.6	28.3	8.3	96.8	<0.001

Abbreviations: OR—odds ratio; CI—confidence interval; BMI—Body Mass Index; PISA—Periodontal Inflamed Surface Area.

## Data Availability

Data are available upon request to the corresponding author.

## Abbreviations

The following abbreviations are used in this manuscript:

LC	Liver Cirrhosis
CLD	Chronic Liver Disease
MELD	Model for End-Stage Liver Disease
INR	International Normalized Ratio
BMI	Body Mass Index
PI	Plaque Index
BoP	Bleeding on Probing
PPD	Probing Pocket Depth
GR	Gingival Recession
CAL	Clinical Attachment Level
PESA	Periodontal Epithelial Surface Area
PISA	Periodontal Inflamed Surface Area
SD	Standard Deviation
IQR	Interquartile Range
OR	Odds Ratio
CI	Confidence Interval
AST	Aspartate Aminotransferase
ALT	Alanine Aminotransferase
GGT	Gamma-Glutamyl Transferase
EFP	European Federation of Periodontology
WONCA	World Organization of Family Doctors
