# Correction: Rinčić et al. Is Periodontal Inflammation Associated with Liver Cirrhosis? A Cross-Sectional Study. *J. Clin. Med.* 2025, *14*, 6616

**DOI:** 10.3390/jcm15052010

**Published:** 2026-03-05

**Authors:** Goran Rinčić, Marija Roguljić, Nives Rinčić, Lucija Virović Jukić, Petar Gaćina, Darko Božić, Ana Badovinac

**Affiliations:** 1 Sestre Milosrdnice University Hospital Centre, Division of Hematology, Department of Internal Medicine, University of Zagreb School of Dental Medicine, 10000 Zagreb, Croatia; grincic@yahoo.com (G.R.); petar.gacina@kbcsm.hr (P.G.); 2 Department of Periodontology, School of Medicine, University of Split, 21000 Split, Croatia; 3 Department of Periodontology, Dental Outpatient Clinic Zagreb, 10000 Zagreb, Croatia; marijarog@gmail.com; 4 Division of Gastroenterology and Hepatology, Department of Internal Medicine, Sestre Milosrdnice University Hospital Centre, 10000 Zagreb, Croatia; lucija.virovic.jukic@kbcsm.hr; 5 Department of Periodontology, School of Dental Medicine, University of Zagreb, 10000 Zagreb, Croatia; bozic@sfzg.hr (D.B.); badovinac@sfzg.hr (A.B.)

## Text Correction

In the original publication [1], there were errors in the published version of the article in the Abstract (Results Section) and in the last sentence of the first paragraph of the Discussion, all related to the statistical analysis. In the Abstract, the interquartile range (IQR) of the MELD score was incorrectly reported as 6–33; the correct values are 9–21, as presented in Table 3 of the Results Section. Additionally, the odds ratio values from the logistic regression analysis do not correspond to those shown in Table 4 of the Results Section. Finally, the variable “number of teeth” was incorrectly mentioned as being included in the statistical analysis in the Discussion.

The corrected Abstract and Discussion are provided as follows:

## Abstract

**Background**: Periodontitis is linked to a range of systemic non-communicable diseases, including hepatic diseases. The aim of this study was to investigate whether periodontal health status is associated with liver cirrhosis (LC). **Methods**: Patients were recruited from the Department of Internal Medicine at the University Clinical Hospital “Sestre Milosrdnice” and categorized into two groups. The case group comprised patients with LC, while age-matched individuals without LC served as controls. Systemic health status was evaluated through laboratory tests, medical history, and clinical parameters, and the Model for End-Stage Liver Disease (MELD) score was calculated for each participant. A comprehensive clinical periodontal assessment was conducted, measuring bleeding on probing (BoP), probing pocket depth (PPD), gingival recession (GR), clinical attachment level (CAL), and the Periodontal Inflamed Surface Area (PISA) score. Stepwise logistic regression was employed to assess possible predictors of LC, including periodontal status. **Results**: A total of 100 patients were included in the analysis, consisting of 50 cases with LC and 50 controls. The mean age was 56.79 years (SD = 11.16) of participants, and 58% were male. The majority of LC cases were attributed to alcohol abuse (41/50, 82%), and the median MELD score was 16 (IQR 9–21). Comparison of the two groups revealed significantly worse clinical periodontal parameters in the LC group and a higher prevalence of periodontitis (*p* = 0.012). Among the 50 LC patients, 46 (92%) exhibited severe forms of periodontitis (stages III and IV). Logistic regression analysis identified alcohol consumption (OR = 275.0, 95% CI 52.8–1432.9, *p* < 0.001) and PISA (OR = 28.3, 95% CI 8.3–96.8, *p* < 0.001) as independent predictors of LC. **Conclusions**: Within the limits of the present study, the higher prevalence of periodontal disease in the LC group suggests an association between LC and periodontitis.

## Discussion

The present study comparatively investigated the clinical periodontal and medical parameters in a group of participants with or without liver cirrhosis. The findings in the age test group indicated significantly worse periodontal health and more severe forms of periodontitis in the LC group. The PISA score was found to be an independent predictor of LC, along with alcohol consumption.

## Author Contribution Correction

In the Author Contributions, author Nives Rinčić passed away prior to the publication of this manuscript, which is now stated in the Author Contributions Section. A correction has been made to the Author Contributions.

**Author Contributions:** G.R. performed medical treatment of participants, data collection, and interpretation and writing the manuscript, M.R. participated in data interpretation, performed statistical analysis, and writing the manuscript, N.R. contributed to conceptualization, patient clinical examination, data collection and drafting the manuscript, L.V.J. provided and selected participants and wrote the manuscript, P.G. provided and selected participants and wrote the manuscript, D.B. participated in conceptualization and methodology and writing the manuscript, A.B. participated in conceptualization and methodology, coordination, writing the manuscript and supervision of the whole research. Author Nives Rinčić passed away prior to the publication of this manuscript. All authors have read and agreed to the published version of the manuscript.

The authors state that the scientific conclusions are unaffected. This correction was approved by the Academic Editor. The original publication has also been updated.
